# An accessible visible-light actinometer for the determination of photon flux and optical pathlength in flow photo microreactors

**DOI:** 10.1038/s41598-018-23735-2

**Published:** 2018-04-03

**Authors:** Anca Roibu, Senne Fransen, M. Enis Leblebici, Glen Meir, Tom Van Gerven, Simon Kuhn

**Affiliations:** Department of Chemical Engineering, KU Leuven, Celestijnenlaan 200F, 3001 Leuven, Belgium

## Abstract

Coupling photochemistry with flow microreactors enables novel synthesis strategies with higher efficiencies compared to batch systems. Improving the reproducibility and understanding of the photochemical reaction mechanisms requires quantitative tools such as chemical actinometry. However, the choice of actinometric systems which can be applied in microreactors is limited, due to their short optical pathlength in combination with a large received photon flux. Furthermore, actinometers for the characterization of reactions driven by visible light between 500 and 600 nm (e.g. photosensitized oxidations) are largely missing. In this paper, we propose a new visible-light actinometer which can be applied in flow microreactors between 480 and 620 nm. This actinometric system is based on the photoisomerization reaction of a diarylethene derivative from its closed to the open form. The experimental protocol for actinometric measurements is facile and characterized by excellent reproducibility and we also present an analytical estimation to calculate the photon flux. Furthermore, we propose an experimental methodology to determine the average pathlength in microreactors using actinometric measurements. In the context of a growing research interest on using flow microreactors for photochemical reactions, the proposed visible-light actinometer facilitates the determination of the received photon flux and average pathlength in confined geometries.

## Introduction

Photochemical reactions carried out in flow microreactors are progressively applied in organic synthesis^1^. This is stimulated by the new reaction pathways enabled by photochemistry coupled with the homogeneous illumination of the entire reactor volume and enhanced mass transfer offered by microreactors. The understanding of photochemical reaction mechanisms and their transfer to industry is accelerated when the emission spectrum of the light source, the photon flux absorbed by the reaction mixture and the quantum yield are provided for each study. By reporting the spectrum of the light source and absorbed photon flux, the reproducibility of the photochemical reactions could be improved. For example, the overexposure to radiation when the same reaction is applied in a different experimental set-up can easily be identified and avoided in case it causes secondary reactions and decreased yield^2^. The quantum yield represents the ratio between the number of molecules converted or formed and the number of absorbed photons of a certain wavelength in the same period of time^3^. Knowing the value of the quantum yield can help to understand the reaction mechanism^4^ or to transfer the chemical reaction from laboratory to a larger scale^5^. When the intrinsic kinetic parameters such as quantum yield are available, reactor and process design can be realized by modelling, representing a faster and more cost-effective strategy than the conventional trial-and-error approach. Due to the small dimensions of microreactors, the quantification of the photon flux passing through the reactor space cannot be performed using physical measurement devices. Therefore, the photon flux determination for microreactors relies on chemical actinometry. A chemical actinometer is a chemical system which undergoes a light-driven transformation for which the quantum yield, *ϕ*_*λ*_, is known and “by which the number of photons in a beam absorbed into the defined space of a chemical reactor can be determined integrally or per unit time”^3^. For accurate photon flux determination using chemical actinometry, it is necessary to operate in a regime where conversion changes linearly with the residence time and the actinometer solution is characterized by constant optical properties (absorbance variation below ±10%) during irradiation. If this is not possible, either operation under complete absorption of the incident light or integration of differential absorbance over time is required^3^. These conditions are difficult to fulfill in microreactors, as the high photon densities achieved in the microchannel lead to high conversions, and the short optical pathlength (typically smaller than 0.1 cm) to low absorption of the incident light by the actinometer. A widely accepted actinometer is the Hatchard-Parker actinometer (ferrioxalate) which is characterized by a quantum yield higher than 0.9^6^. It involves the reduction of K_3_Fe(C_2_O_4_)_3_·3H_2_O in the presence of light, and the resulting Fe^2+^ ions are quantified by the complexation with 1,10-phenanthroline. Using the ferrioxalate actinometer, Aillet *et al*.^7^ reported difficulties in the characterization of microreactors as the large concentrations required to ensure full absorption lead to the formation of solids. Furthermore, reaching the regime where conversion changes linearly with residence time required large flowrates, exceeding the design specifications of available pumps to overcome the pressure drop in the microchannels. Moreover, the applicability of this actinometer system lies in the 250 nm to 500 nm range. When the light source emission exceeds 500 nm, the photon flux can be quantified using meso-diphenylhelianthrene^3,8^, an in-house synthesized diarylethene derivative actinometer (1,2-bis(5-(4-ethynylphenyl)-2-methylthiophen-3-yl)perfluorocyclopentene)^9^, Reinecke salt^3,10–12^, Aberchrome 540^3,13^ or 1,9-diphenylanthracene (DPA)^4^, their characteristics are presented in Table 1.

**Table 1:** Characteristics of commonly used visible-light actinometers (for spectral range higher than 500 nm) and of the proposed actinometer system.

Actinometer	Spectral range nm	Φ	Commercial availability	Preparation/Analysis
Meso-diphenylhelianthrene^3,8^	475–610	0.224	No	−/Absorbance at 429 nm
1,2-Bis(5-(4-ethynylphenyl)-2-methylthiophen-3-yl) perfluorocyclopentene^9^	400–620	0.016–0.0015	No	Irradiation with UV light/Absorbance at 600 nm
Reinecke salt^3,10–12^	316–750	~0.3	Yes	Conversion of NH_4_[Cr(NH_3_)_2_(CNS)_4_] to potassium salt/Absorbance at 450 nm of Fe(III) thiocyanate complex
Aberchrome 540^3,13^	436–546	0.073–0.047	Yes	Irradiation with UV light/Absorbance at 494 nm or 343 nm
DPA^4^	400–550^a^	0.019	Yes	−/Absorbance at 372 nm
Proposed actinometer (1,2-bis(2,4-dimethyl-5-phenyl-3-thienyl)perfluorocyclopentene)^18^	480–620	0.027–0.015	Yes	Irradiation with UV light/Absorbance at 562 nm

^a^Absorption range of Ru(bpy)_3_Cl_2_ in acetonitrile.

**Figure 1:**
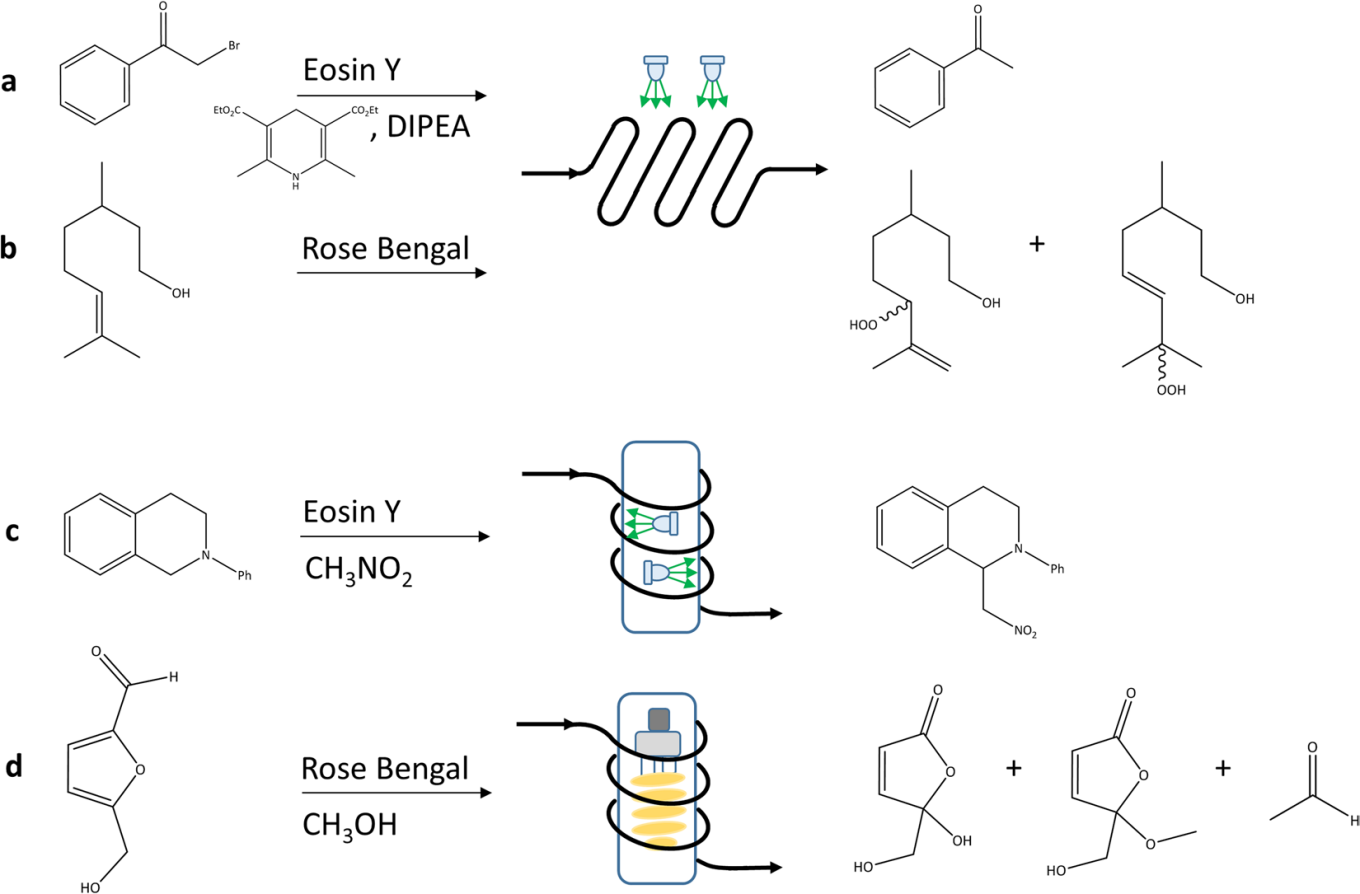
Examples of continuous flow photochemical reactions driven by visible light between 500 and 600 nm. (**a**) Reductive dehalogenation of 2-bromoacetophenone photocatalyzed by Eosin Y carried out in a microstructured reactor under irradiation by green LEDs. (DIPEA: diisopropylethylamine)^14^. (**b**) Oxidation of citronellol photosensitized by Rose Bengal carried out in a microstructured reactor under irradiation by green LEDs^15^. (**c**) Oxidative coupling reaction of N-phenyl tetrahydroisoquinoline with nitromethane photocatalyzed by Eosin Y under irradiation by green LEDs carried out in a capillary reactor^16^. (**d**) Oxidation of 5-(hydroxymethyl)furfural photosensitized by Rose Bengal under irradiation by a fluorescent light source carried out in a capillary reactor^17^.

As it can be observed, only Reinecke salt, Aberchrome 540 and DPA actinometers are based on commercially available starting materials. However, Reinecke salt involves a complex experimental procedure and a dark thermal reaction. Aberchrome 540 involves a simple handling of the actinometer solution, but the quantum yields are only reported until a wavelength of 546 nm^3^. Recently, a new visible-light actinometer which uses the singlet oxygen oxidation of 1,9-diphenylanthracene (DPA) in the presence of Ru(bpy)_3_Cl_2_ photocatalyst was reported^4^. However, the applicability range of this actinometer is limited by the catalyst absorption (estimated between 400 and 550 nm in acetonitrile). Therefore, photochemical reactions which are carried out in the presence of Rose Bengal (λ_max_ = 559 nm in methanol) or Eosin Y (λ_max_ = 539 nm in acetonitrile) and under visible light irradiation ranging between 500 and 600 nm^14–17^ (see Fig. 1) cannot be characterized using Aberchrome 540 and DPA actinometers.

Sumi *et al*.^18^ reported the quantum yield for the cycloreversion reaction of two diaryethenes derivatives which absorb in the visible range: 1,2-bis(2-methyl-1-benzothiophen-3-yl)perfluorocyclopentene (*ϕ* around 0.3 for 450-580 nm) and 1,2-bis(2,4-dimethyl-5-phenyl-3-thienyl)perfluorocyclopentene (*ϕ* around 0.02 for 480–620 nm). The cycloreversion reaction involves the isomerization between a closed form which absorbs visible light to an open form which absorbs UV light. The cyclization reaction from the open to the closed form is driven by UV radiation. In the present work, we propose 1,2-bis(2,4-dimethyl-5-phenyl-3-thienyl)perfluorocyclopentene as new visible-light actinometer suitable for the photon flux quantification in flow microreactors due to the following reasons: (i) the low quantum yield of the visible light-driven reaction makes the system suitable for performing actinometric measurements in microreactors; (ii) it covers a wide range of the visible region between 480 and 620 nm; (iii) the high quantum yield (0.45) of the isomerization from the open to the closed form under UV irradiation enables a fast generation of the compound necessary for the actinometric measurements.

As the dependence of the quantum yield on wavelength is known between 480 and 620 nm, the proposed system can also be used for actinometric studies with polychromatic light sources^18^. In addition, it is a thermally stable compound^19,20^ and it was reported to undergo the cyclization/cycloreversion reaction for around 30000 cycles in solid phase^21^. As the kinetic model which characterizes the isomerization reaction from closed to open form was extensively studied, this new visible-light actinometer offers the possibility to calculate the photon flux with an analytical solution, even when the conversion exceeds the linear region^22,23^.

In this study, we implement a new visible-light actinometer based on 1,2-bis(2,4-dimethyl-5-phenyl-3-thienyl)perfluorocyclopentene for measurements in flow microreactors. We describe the experimental procedure for conducting actinometric measurements in a microreactor illuminated by green Light Emitting Diodes (LEDs) and equipped with on-line UV-Vis absorption analysis. The procedure to calculate the photon flux and the conditions of collimated and monochromatic light are extensively discussed. Moreover, the importance of using the correct optical pathlength in the calculations of the photon flux is proven.

## Results and Discussion

### Optical properties of DAE solutions and protocol for actinometric measurements

For simplifying the descriptions we will use DAE to refer to 1,2-bis(2,4-dimethyl-5-phenyl-3-thienyl)perfluorocyclopentene. The flow microreactor used in this study is characterized by an optical pathlength in the order of 0.1 cm (equaling the diameter of the circular microchannels), which results in high photon fluxes throughout the reactor channel. Consequently, to avoid complete or high conversions, concentrated solutions of the closed form DAE (DAE CF) up to 10^−3^ M are used in this work in comparison with 10^−5^ M used previously^18,20^ for determination of the optical properties of the system. A solution of pure open form DAE (DAE OF) and a mixture of isomers after irradiation with a 311 nm UV lamp were analyzed with a UV-Vis flow cell connected to a spectrometer. A linear variation of the absorbance with concentration is obtained as long as the absorbance is below 1 (see Supplementary Fig. S2), which was then defined as absorbance limit for all experiments.

**Figure 2:**
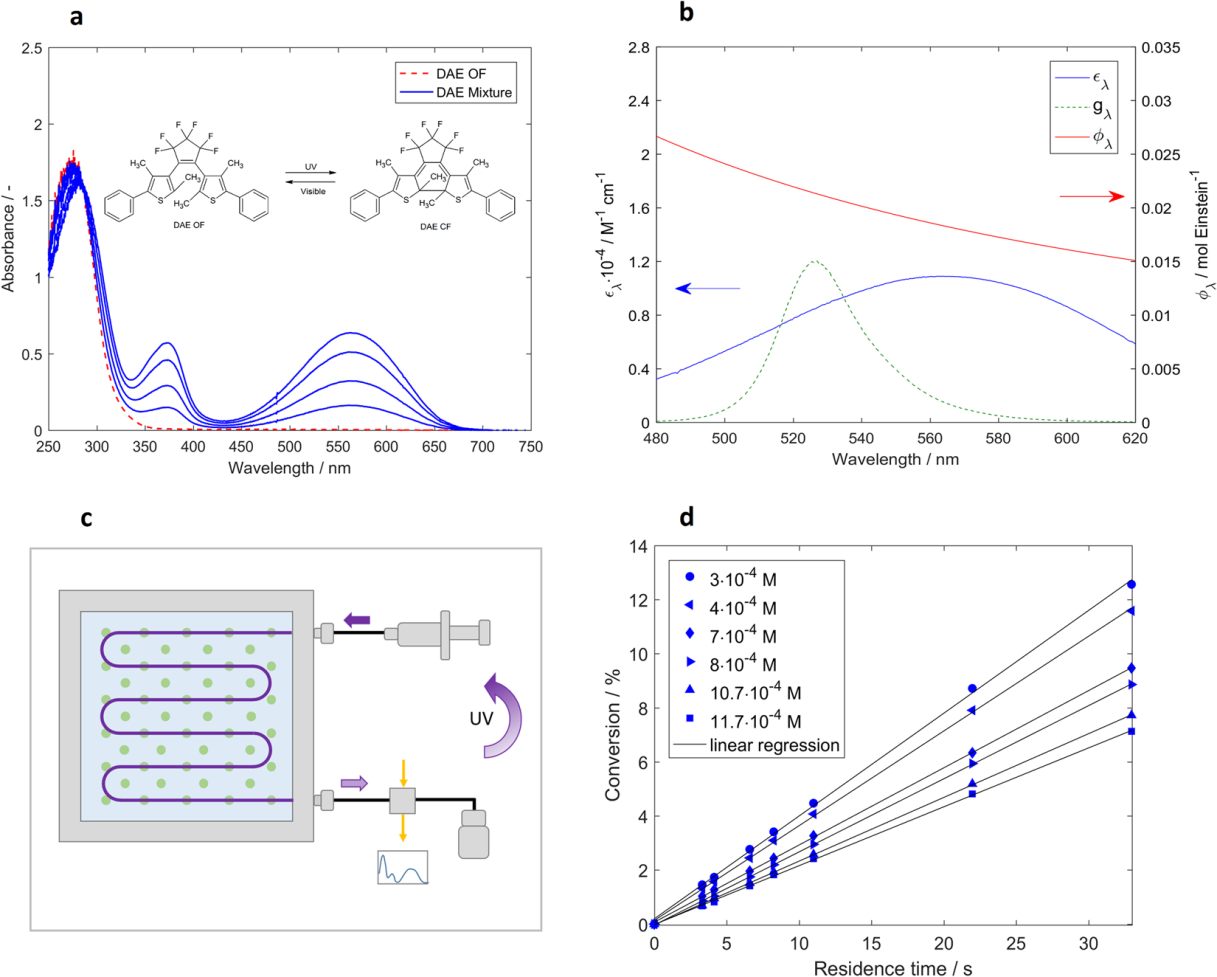
Optical properties of the actinometer system and experimental set-up. (**a**) Absorption spectra of the open form DAE (DAE OF) solution in hexane before irradiation and of DAE OF and closed form (DAE CF) mixture after UV irradiation at increasing residence times. *λ*_max_ of the UV lamp is 311 nm. (**b**) Variation of DAE CF molar absorption coefficient, *ε*_*λ*_, and of cycloreversion reaction quantum yield, *ϕ*_*λ*_, in the range of LED emission (illustrated through the LED energy density distribution function, *g*_*λ*_). (**c**) Scheme of the experimental set-up for performing actinometric measurements. The LED light source (indicated by green spots) is positioned behind the microreactor at a specified distance, *L*, from the microreactor. (**d**) Variation of the conversion of DAE CF solutions with different initial concentrations subject to residence time (*L* = 2 cm, *I*_F_ = 2.76 mA/LED).

The absorption spectra of DAE OF and DAE CF in hexane were found to be similar with the ones reported by Sumi *et al*.^18^ As it can be observed in Fig. 2a, DAE OF exhibits an absorption peak in the UV region with the maximum at 268 nm. Upon irradiation of DAE OF with a 311 nm UV lamp DAE CF is formed, which is identified by the additional absorption peaks developing at 370 nm and 562 nm. The absorption spectra of the two isomers mixture are characterized by an isosbestic point at 284 nm which proves that no side product with different optical properties was formed during the UV irradiation. To achieve high conversion towards the DAE CF, the irradiation time was increased by passing the actinometer solution multiple times through the UV capillary reactor (a description of the UV capillary reactor is provided in the Supplementary Information section S4). When the conversion from DAE OF to DAE CF reached around 70% the irradiation was stopped. The reported conversion value in the photostationary state under irradiation with a wavelength of 313 nm is 79%^20^.

The molar absorption coefficient of DAE OF was determined to be 28381 ± 248 M^−1^cm^−1^ (three replicates) at 268 nm which is close to the value of 28400 M^−1^cm^−1^ reported by Sumi *et al*.^18^. The determination of the molar absorption coefficient of DAE CF cannot be realized using the conventional procedure based on known concentrations, as this isomer is not commercially available and as outlined above the OF to CF conversion upon UV irradiation is not complete in the photostationary state. In this study, the highest employed concentration of DAE CF is 1.2·10^−3^ M. This starting solution was sequentially diluted and a linear variation of the absorbance with concentration was observed. Hence, a molar absorption coefficient of 10900 M^−1^cm^−1^ at 562 nm reported by Sumi *et al*.^18^ and determined at concentrations in the order of 10^−5^ M was used further in this study.

As illustrated in Fig. 2c, the procedure for performing actinometric measurements using DAE solutions is simpler than in the case of the ferrioxalate actinometer and it involves two steps: (i) conversion of DAE OF to DAE CF under UV irradiation (λ_max_ of 311 nm) (ii) conversion from DAE CF to the DAE OF in the photoreactor under irradiation with visible light.

The glass plate microreactor was placed at a distance, *L*, of 2 cm from the LED light source, which was set to a forward current, *I*_F_, of 2.76 mA per LED. The solutions formed upon irradiation in the UV capillary reactor were fed through the microreactor at flow rates ranging from 1 to 10 ml min^−1^ which corresponds to residence times between 3.3 and 33 s. The concentration of the actinometric solution exiting the reactor was determined from absorbance measurements using a UV-Vis flow cell connected to the outlet of the reactor. For this, the interval of 565–566 nm was continuously monitored, but only the values after the absorbance became constant were considered in the photon flux calculations. We have waited for at least 7 residence times before recording the absorbance for at least 20 seconds. The lowest flow rate of 1 ml min^−1^ was chosen to ensure that the residence time in the flow cell is short and the reaction in the flow cell is negligible for the overall conversion.

We conducted actinometric measurements at the same operating conditions (flow rate and light intensity) using initial concentrations of DAE CF comprised between 3 and 12·10^−4^ M. As it can be observed in Fig. 2d, the conversion of DAE CF increases linearly with residence time. The maximum conversion of 12.5% was achieved in the case of 3·10^−4^ M solutions. The linear regression is characterized by a R^2^ value which varied between 0.9966 at 3·10^−4^ M to 0.9998 at 1.2·10^−3^ M.

### Calculation of the photon flux

The variation of the concentration, *c* [M], of the closed form isomer with the irradiation time (equaling the residence time in a flow reactor), *t* [s], upon irradiation with monochromatic light in the visible region is determined by the quantum yield, *ϕ*_*λ*_ [mol Einstein^−1^] of the cycloreversion reaction and the absorbed photon flux density, *L*_abs,0_ [Einstein L^−1^ s^−1^], averaged over the illuminated volume as shown in equation (1). The absorbed photon flux *I*_abs,λ_ [Einstein s^−1^] can be calculated from *L*_abs,λ_ by multiplying it with the irradiated volume, *V* [L].

1 $$\frac{{\rm{d}}c}{{\rm{d}}t}=-{{\varphi }}_{\lambda }{L}_{{\rm{abs}},\lambda }=-{{\varphi }}_{\lambda }\frac{{I}_{{\rm{abs}},\lambda }}{V}$$

The objective in actinometric measurements is to determine the overall received photon flux in the channel (*I*_0,λ_) irrespective of the photon flux absorbed by the actinometric solution (*I*_abs,λ_). When operating in the complete absorption regime, the two fluxes are equal, *I*_0,__λ_ = *I*_abs,λ_. As it is difficult to absorb all the received light in microreactors, the Lambert-Beer law is used to calculate the transmitted photon flux (*I*_trans,λ_) according to:

2 $${T}_{{\lambda }}=\frac{{I}_{{\rm{trans}},\lambda }}{{I}_{0,\lambda }}={10}^{-{A}_{{\lambda }}}\,{\rm{with}}\,{A}_{{\lambda }}={{\varepsilon }}_{\lambda }c{l}_{{\rm{reactor}}}$$

3 $${I}_{{\rm{abs}},{\lambda }}={I}_{0,{\lambda }}-{I}_{{\rm{trans}},{\lambda }}={I}_{0,{\lambda }}(1-{10}^{-{{\varepsilon }}_{{\lambda }}c{l}_{{\rm{reactor}}}})$$

where *T*_λ_ represents the transmittance at the irradiation wavelength, *A*_λ_ is the solution absorbance at the irradiation wavelength, *ε*_*λ*_ [M^−1^ cm^−1^] is the molar absorption coefficient of the absorbing species at the irradiation wavelength and *l*_reactor_ [cm] is the pathlength of the light in the photoreactor.

The absorbed photon flux *I*_abs,λ_ in equation (1) can be expressed as a function of the received photon flux (equation (3)), which then results in:

4 $$\frac{{\rm{d}}c}{{\rm{d}}t}=-{{\varphi }}_{{\lambda }}\frac{{I}_{0,{\lambda }}}{V}(1-{10}^{-{{\varepsilon }}_{{\lambda }}c(t){l}_{{\rm{reactor}}}})$$

As it can be observed from equation (4), the evolution of the reaction is influenced by the concentration of the absorbing species which leads to the differential equation:

5 $$\frac{{\rm{d}}c}{1-{10}^{-{\varepsilon }_{\lambda }c(t){l}_{{\rm{r}}{\rm{e}}{\rm{a}}{\rm{c}}{\rm{t}}{\rm{o}}{\rm{r}}}}}=-{\varphi }_{\lambda }\frac{{I}_{0,\lambda }}{V}{\rm{d}}{\rm{t}}$$

This differential equation is difficult to solve analytically, however, for the case of unimolecular photoreactions, an analytical solution was previously reported, and the variation of the concentration can be calculated as^18,22,23^:

6 $$\mathrm{log}\,({10}^{{{\varepsilon }}_{{\lambda }}c(0){l}_{{\rm{reactor}}}}-1)-\,\mathrm{log}\,({10}^{{{\varepsilon }}_{{\lambda }}c(t){l}_{{\rm{reactor}}}}-1)={{\varepsilon }}_{{\lambda }}{l}_{{\rm{reactor}}}{{\varphi }}_{{\lambda }}\frac{{I}_{0,{\lambda }}}{V}t$$

Equation (6) can be applied when working with collimated light, monochromatic radiation, and perfectly mixed reaction medium. It was previously used by Sumi *et al*.^18^ to determine the quantum yield of the cycloreversion reaction at 22 °C. They found that the quantum yield depends on the irradiation wavelength as shown by equation (7) and illustrated in Fig. 2c.

7 $${\rm{l}}{\rm{o}}{\rm{g}}\,\varphi =-2.67+\frac{526}{\lambda },\,{\rm{w}}{\rm{i}}{\rm{t}}{\rm{h}}\,480\,{\rm{n}}{\rm{m}}\,\le \,\lambda \,\le \,620\,{\rm{n}}{\rm{m}}$$

#### Collimated vs. uncollimated light

**Figure 3:**
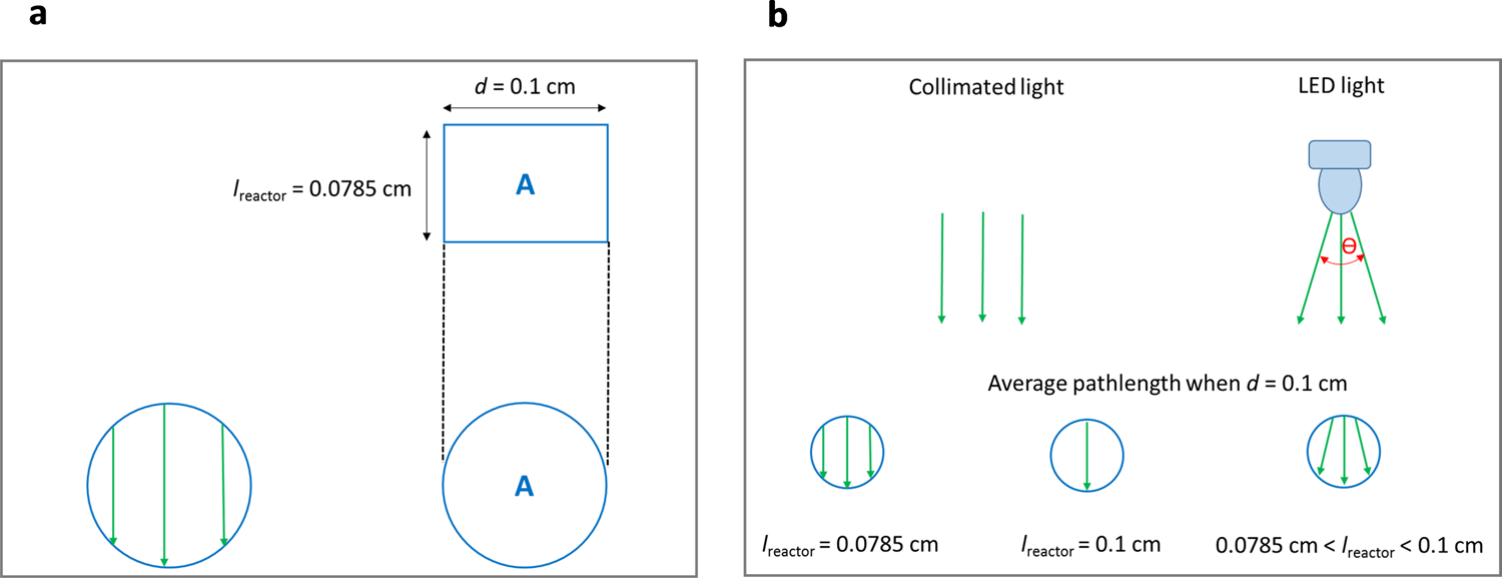
Pathlength estimation. (**a**) The principle of pathlength estimation for a capillary characterized by a circular cross-section and a diameter of 0.1 cm resulting to 0.0785 cm (using the approach reported by Russo *et al*.^25^). (**b**) Common assumptions for pathlength estimation in circular tubes.

The collimated light condition assumes that the incident light consists of parallel rays. In this study, we will consider that the rays are not significantly refracted at the air-glass and glass-liquid boundaries. In the case of channels with circular cross section, two assumptions are commonly used to estimate the optical pathlength. In the first assumption, the pathlength is estimated to be equal to the internal diameter of the channel, *d*. This estimation was used in the case of a light beam narrower than *d*, i.e. analytical flow cells^24^, or when the geometry of the reactor channel was approximated to two parallel plates^7^. In the second assumption, the distance travelled by each ray depends on its entry point relative to the center of the circle. Consequently, the pathlength will equal the diameter of the channel when traversed at the center, and will decrease with increasing offset when the rays pass through the sides of the circular cross-section as illustrated in Fig. 3a. An average pathlength can be calculated based on the following procedure^25^: One can construct a rectangle with an area equal to the capillary cross-section, with the length of one side equal to the capillary diameter and the other to the average pathlength^25^. Therefore, *l*_reactor_ is considered as an average pathlength which can be estimated as the ratio between the area (*A*) and diameter of the channel (*d*).

8 $${l}_{{\rm{reactor}}}=\frac{A}{d}$$

When dealing with LED light sources, the radiation pattern will depend on the viewing angle, θ, as illustrated in Fig. 3b. As our LED light source is built with LEDs with a narrow viewing angle of 30°, we initially assume collimated light and consider the pathlength of 0.0785 cm calculated using equation (8).

#### Monochromatic vs. polychromatic light

In photochemical reactors, the irradiation is often polychromatic, even when using recently developed LED light sources, which are in general characterized by a full width at half maximum (FWHM) exceeding 20 nm. As illustration, the spectral distribution of the LED emission used in this work with a FWHM of 29 nm is depicted in Fig. 2b.

Photons with different energies will contribute to the conversion of DAE CF, in a proportion given by the energy density distribution function of the LED light source, *g*_*λ*_. In this study, *g*_*λ*_ was experimentally determined for each applied forward current from the ratio between the spectral irradiance *E*_*λ*_ (W cm^−2^ nm^−1^) and the sum of spectral irradiances over all emitted wavelengths with a wavelength discretization of 0.3 nm according to:

9 $${g}_{{\lambda }}=\frac{{E}_{{\lambda }}}{\sum _{{\lambda }=480}^{620}{E}_{{\lambda }}}$$

The spectral irradiance, *E*_*λ*_, was measured using a cosine corrector coupled to an optical fiber and a spectrometer. Considering this definition of *g*_*λ*_, equation (4) can be rewritten as a sum of monochromatic contributions averaged by *g*_*λ*_.

10 $$\frac{{\rm{d}}c}{{\rm{d}}t}=-\frac{{I}_{0}}{V}\sum _{{\lambda }=480}^{620}{{\varphi }}_{{\lambda }}{g}_{{\lambda }}(1-{10}^{-{{\varepsilon }}_{{\lambda }}c(t){l}_{{\rm{reactor}}}})$$

The resulting differential equation (10) usually has to be integrated numerically^7^. The procedure for the numerical integration is presented in the Supplementary Information (section S7) and the calculated photon fluxes using a pathlength of *l*_reactor_ = 0.0785 cm are listed in Table 2. The small standard deviations found for the photon fluxes prove the excellent reproducibility of the actinometric measurements.

**Table 2:** Photon flux determined for different concentrations of DAE closed form (DAE CF) (*l*_reactor_ = 0.0785 cm, *L* = 2 cm, *I*_F_ = 2.76 mA/LED).

DAE CF Concentration·10^4^ M	ε_avg_ *cl*_reactor_	*I*_0_ · 10^8^ Einstein s^**−**1^	
		Analytical	Numerical
3.04	0.21	8.61 ± 0.12^a^	8.75 ± 0.12^a^
4.28	0.29	8.45	8.59
7.24	0.50	8.24	8.37
8.34	0.57	8.25 ± 0.10^a^	8.38 ± 0.10^a^
10.70	0.74	8.18	8.30
11.74	0.81	8.18 ± 0.15^a^	8.30 ± 0.16^a^

^a^Average value and standard deviation calculated for 4 replicates.

However, to improve on the ease of implementation, we propose an analytical approximation which in its final form resembles the equation for the case of monochromatic light, but instead considers the weight-averaged optical properties.

11 $$\mathrm{log}\,({10}^{{{\varepsilon }}_{{\rm{avg}}}c(0){l}_{{\rm{reactor}}}}-1)-\,\mathrm{log}\,({10}^{{{\varepsilon }}_{{\rm{avg}}}c(t){l}_{{\rm{reactor}}}}-1)={{\varepsilon }}_{{\rm{avg}}}{l}_{{\rm{reactor}}}{{\varphi }}_{{\rm{avg}}}\frac{{I}_{0}}{V}t$$

with

12 $${{\varepsilon }}_{{\rm{avg}}}=\sum _{{\lambda }=480}^{620}{{\varepsilon }}_{{\lambda }}{{g}}_{{\lambda }}$$

13 $${{\varphi }}_{{\rm{avg}}}=\sum _{{\lambda }=480}^{620}{{\varphi }}_{{\lambda }}{g}_{{\lambda }}$$

As shown in the Supplementary Information (section S8), equation (11) results from considering that the first term in an asymptotic expansion, *δ*_*λ*_, is a small number:

14 $${{\delta }}_{{\lambda }}=\frac{{{\varepsilon }}_{{\lambda }}}{\sum _{{\lambda }=480}^{620}{{\varepsilon }}_{{\lambda }}{g}_{{\lambda }}}-1$$

The definition of *δ*_*λ*_ contains the ratio of the molar absorption coefficients ε_λ_ corresponding to the LED emission and the weight-averaged value of *ε*_*λ*_, Σ*ε*_*λ*_*g*_*λ*_. In the case of monochromatic light, this ratio is equal to 1 and *δ*_*λ*_ equal to 0. In our experimental conditions with polychromatic light, $|{{\delta }}_{{\lambda }}|$ is lower than 0.63.

For calculating the photon flux using the analytical estimation, the left hand side of equation (11) (in the following denoted by *S*) is plotted as a function of the residence time. A linear curve is obtained with the slope equal to ${{\varepsilon }}_{{\rm{avg}}}{l}_{{\rm{reactor}}}{{\varphi }}_{{\rm{avg}}}\frac{{I}_{0}}{V}$. The obtained linear regressions are characterized by coefficients R^2^ higher than 0.9996. Considering an optical pathlength of *l*_reactor_ = 0.0785 cm, the photon flux *I*_*0*_ was calculated from the slope and is listed in Table 2.

By comparing the photon fluxes listed in Table 2, it can be observed that the analytical estimation slightly underestimates the values of the photon flux. The differences between the photon flux determined by the analytical estimation and numerical integration increased from 1.45 to 1.60% with decreasing concentration.

**Figure 4:**
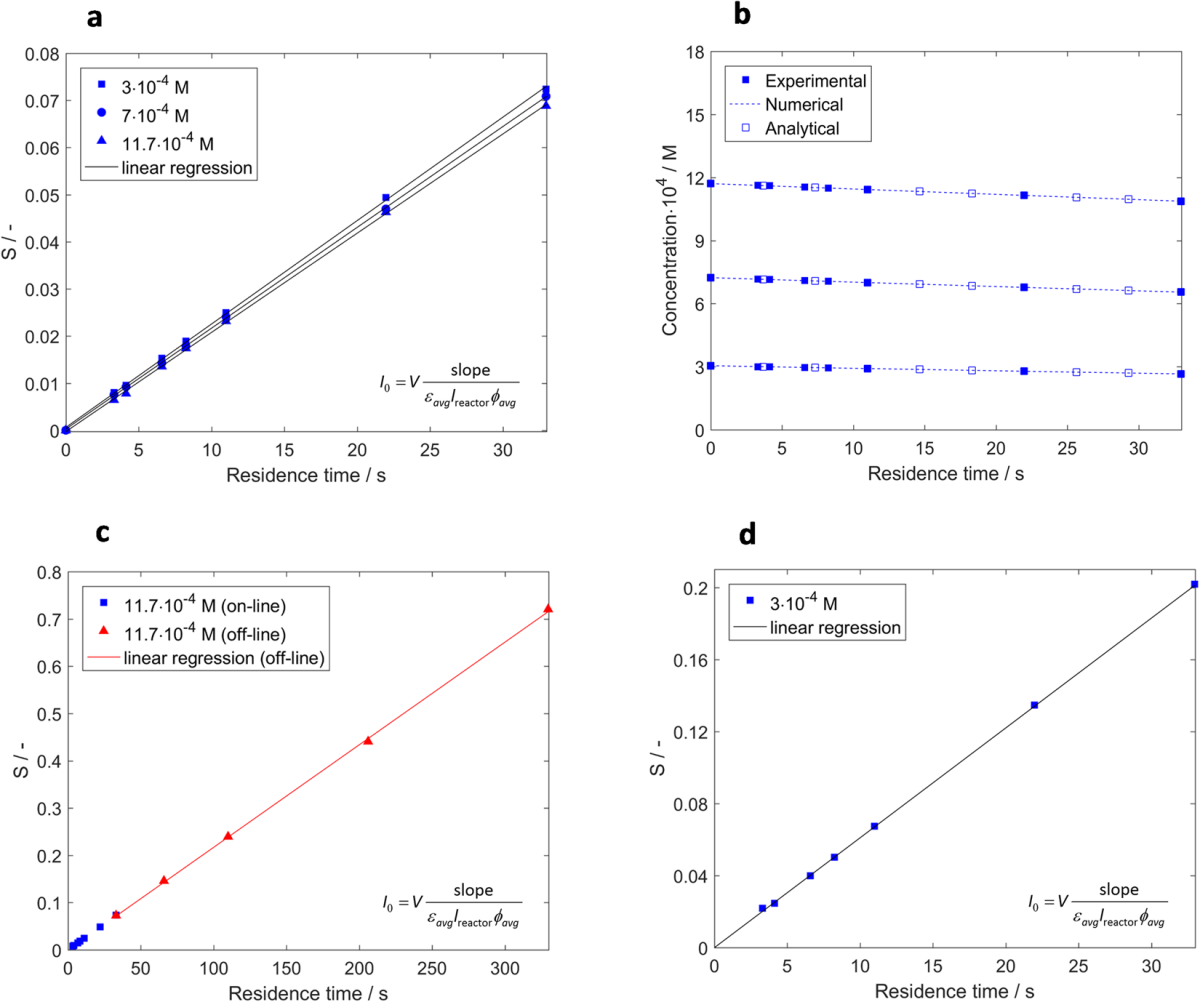
Evolution of DAE CF concentration during continuous irradiation with green LEDs: experimental data and predictions. (**a**) Variation of *S* as a function of residence time at different initial concentrations (*l*_reactor_ = 0.0785 cm, *L* = 2 cm, *I*_F_ = 2.76 mA/LED). (**b**) Variation of DAE CF concentration as a function of residence time at different initial concentrations. The experimental data are compared with the results of numerical integration and analytical estimation (*l*_reactor_ = 0.0785 cm, *L* = 2 cm, *I*_F_ = 2.76 mA/LED). (**c**) Variation of *S* as a function of residence time between 3 and 330 s at 11.7·10^−4^ M. The experimental data acquired at residence times of 33–330 s were obtained by off-line analysis (*l*_reactor_ = 0.0785 cm, *L* = 2 cm, *I*_F_ = 2.76 mA/LED). (**d**) Variation of *S* as a function of residence time at 11.7·10^−4^ M and *I*_F_ = 8.1 mA/LED (*l*_reactor_ = 0.0785 cm, *L* = 2 cm).

The determined photon fluxes were used to predict the variation of DAE CF with residence time, the profiles being in a good match with the experimental data (see Fig. 4b). Furthermore, we performed the actinometric measurements at longer residence times in the photo microreactor and at higher LED intensity. Actinometric solutions with concentration of 11.7·10^−4^ M were irradiated at residence times between 33 and 330 s (flow rates between 0.1 and 1 ml min^−1^). As previously described, the on-line analysis could not be used for flow rates smaller than 1 ml min^−1^. In this case, samples were collected and analyzed off-line using the flow cell. As it can be seen in Fig. 4c, *S* varies linearly with residence time between 33 and 330 s, even if a high conversion of 62% is reached. The linear regression is characterized by a coefficient R^2^ of 0.9997. The photon flux determined analytically for the residence time interval of 33–330 s was found to be 8.31·10^−8^ Einstein s^−1^ and it is similar with the photon flux determined at 3–33 s (8.18 ± 0.15·10^−8^ Einstein s^−1^). In the former case, the difference between the photon flux determined by analytical estimation and numerically is 1.42%. In addition, we have performed actinometric measurements at 3·10^−4^ M and at higher forward current, *I*_F_, of 8.1 mA/LED at residence time between 3 and 33 s (see Fig. 4d). The maximum conversion of 31.8% was achieved after 33 s. The photon flux determined analytically is 2.37·10^−8^ Einstein s^−1^ and is 1.25% lower than the value obtained by numerical integration. The linearity of *S* vs. residence time over a wide range of operation conditions and the good prediction of the concentration variation with the residence time show that the actinometric measurements performed in our microreactor are not affected by diffusion limitations (see Supplementary Information section S9 for an extended discussion about diffusion limitations in flow microreactors).

#### Influence of average optical pathlength

**Figure 5:**
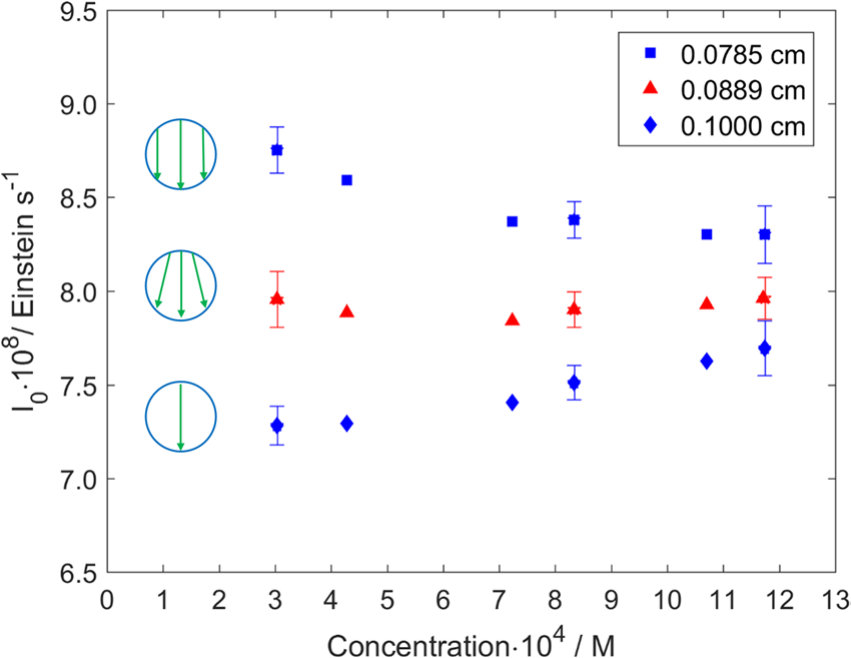
Variation of the photon flux, *I*_0_, with the closed form DAE (DAE CF) concentration. Three limiting cases are considered: parallel rays covering the channel section (*l*_reactor_ = 0.0785 cm), non-parallel rays covering the channel section (*l*_reactor_ = 0.0889 cm) and parallel rays passing only through the center of the channel (*l*_reactor_ = 0.1 cm). The photon flux is calculated numerically from the experimental data acquired at *L* = 2 cm and *I*_F_ = 2.76 mA/LED. The error bars show the standard deviation calculated for 4 replicates.

The photon fluxes reported in Table 2 appear to decrease with increasing DAE CF concentration regardless of the calculation method. Variations of the quantum yield or the refractive index might be a probable cause for this observation. However, upon further evaluation these factors were excluded as we observed this trend is caused by the chosen value for the optical pathlength (see Fig. 5).

Considering an average pathlength equal to the diameter of the reactor channel, a reverse trend, i.e. increase of the photon flux with actinometer concentration is observed. These observed trends cannot be valid, as a constant photon flux should be achieved when the correct average pathlength is used in the calculations. Using a smaller average pathlength than the correct value will lead to an overestimation of the received photon flux, and a higher average pathlength will lead to an underestimation of the received photon flux. As it can be seen in Fig. 5, the degree of over- and underestimation is minimized when performing actinometric measurements at higher concentrations. This is determined by the term $(1-{10}^{-{{\varepsilon }}_{{\lambda }}c(t){l}_{{\rm{reactor}}}})$ in equation (10) which approaches the value of 1 at high concentrations. In this limiting condition (complete absorption), the variation of actinometer concentration with residence time becomes independent of the optical pathlength, *l*_reactor_.

Therefore, we further developed a procedure to extract the correct average pathlength from our measurements acquired at different concentrations (including the replicates). Using equation (10), and making use of the fact that the incident photon flux *I*_0_ is constant for all cases, results in a non-linear system of equations. For this numerical solution, the pathlength was varied between 0.07 and 0.1 cm in steps of 0.0001 cm, and a single pathlength value of 0.0889 cm was identified which leads to a minimum difference between photon fluxes (see Table 3). This finding shows that the rays are passing through the entire circular cross-section of the reactor channel and their trajectory is slightly deviating from fully collimated light. Using the so obtained pathlength of *l*_reactor_ = 0.0889 cm for the calculation of the photon flux, the difference in photon fluxes between the highest and least concentrated solution reduced from around 5% (in the case of *l*_reacto__r_ = 0.0785 cm and *l*_reactor_ = 0.1 cm) to an insignificant difference as illustrated in Fig. 5 and Table 3. As an alternative, the pathlength can be calculated similarly from a system of equations using the analytical estimation (equation (11)). The so identified pathlength of 0.0882 is 0.8% smaller in comparison with the pathlength calculated using numerical integration.

**Table 3:** Photon flux determined for different concentrations of DAE closed form (DAE CF). I_0_ was determined numerically using *l*_reactor_ = 0.0889 cm and analytically using *l*_reactor_ = 0.0882 cm. (*L* = 2 cm, *I*_F_ = 2.76 mA/LED).

DAE CF Concentration·10^4^ M	ε_avg_*cl*_reactor_	*I*_0_ · 10^8^ Einstein s^**−**1^	
		Analytical	Numerical
3.04	0.21	7.87 ± 0.12^a^	7.95 ± 0.13^a^
4.28	0.29	7.79	7.88
7.24	0.50	7.74	7.84
8.34	0.57	7.81 ± 0.09 ^a^	7.90 ± 0.10^a^
10.70	0.74	7.84	7.92
11.74	0.81	7.87 ± 0.15^a^	7.96 ± 0.15^a^

^a^Average value and standard deviation calculated for 4 replicates.

## Conclusions

The implementation of a new actinometer system suitable for the visible region between 480 and 620 nm was presented. This system showed excellent reproducibility, uses on-line absorbance measurements and is suitable for a wide range of operation conditions. Two methods to calculate the photon flux were presented and compared: numerical integration and an analytical estimation. The numerical integration was considered as reference due to its higher accuracy. We found that the analytical estimation, which considers weight-averaged optical properties, underestimates the photon flux by a maximum of 1.6%. Moreover, we observed that the considered average optical pathlength is crucial for a correct interpretation of the actinometric results. Therefore, a methodology for experimental determination of the average pathlength in microreactors was proposed. We expect that the use of this actinometer system will significantly contribute to the study of photochemical reactions driven by visible light as it enables researchers to quantitatively characterize their individual photochemical system in terms of photon flux, quantum yield and average pathlength. Moreover, the proposed actinometer could facilitate the investigation of scattering phenomena in complex reactor geometries and a comparative assessment of the efficiencies of different visible-light sources.

## Methods

### Actinometry

DAE CF solution is obtained by irradiating with a UV lamp (PL-S 9 W/01/2P, Philips) with a main emission peak at 311 nm in continuous flow of DAE OF (TCI Chemicals) solution in hexane (Biosolve). The UV reactor is a capillary tube (PFA) with an internal diameter of 0.1 cm. The range of the employed flow rates is 0.5 ml min^−1^ to 1 ml min^−1^. The resulting solution containing a mixture of DAE CF and DAE OF is fed through a plate glass microreactor (Chemtrix Inc.) using a syringe pump (Model Fusion 720, Chemyx Inc.). For achieving flow rates smaller than 1 ml min^−1^, a syringe pump from Harvard Apparatus, PHD ULTRA, was employed. The microreactor consists of a serpentine channel with an internal diameter of 0.1 cm and an overall length of 69.95 cm etched in a borosilicate glass plate. The liquid exiting the reactor flows through a cross-cell (Avantes) for on-line analysis. A balanced Deuterium-Halogen light source (215–2500 nm, Avantes) and a compact spectrometer (Ultra Low Straylight Fiber Optic UV/VIS/NIR spectrometer 200–1100 nm, Avantes) are connected to the flow cell through optical fibers (400 μm UV/VIS fiber, Avantes). The spectrometer is connected to a computer which continuously monitors the absorbance at 565–566 nm (software Avasoft 8). The absorbance measurements are characterized by an integration time of 1.31 ms and 500 scans/measurement. The solution is collected at the outlet of the flow cell and then reused with or without additional UV irradiation. The light source is a LED board connected to a driving board, manufactured in-house at the Department of Chemical Engineering, KU Leuven. The LED board contains 144 LEDs. The used LEDs (HLMP-CM3A-Z10DD, Avago) are characterized by a viewing angle of 30°, a maximum emission at λ_max_ = 527 nm and a FWHM of 29 nm measured at 2.76 mA.

### Data availability statement

The datasets generated during and/or analysed during the current study are available from the corresponding author on reasonable request.

## Electronic supplementary material

Supplementary Information
