# Supplementary material for: An accessible visible-light actinometer for the determination of photon flux and optical pathlength in flow photo microreactors
Source: Sci Rep. 2018 Apr 3;8:5421. doi: 10.1038/s41598-018-23735-2 (PMC5882931; doi:10.1038/s41598-018-23735-2)
Supplement: Supplementary file 1 — Supplementary Information [file 41598_2018_23735_MOESM1_ESM.pdf]

Supplementary Information

An accessible visible-light actinometer for determination of photon flux and optical pathlength in flow photo microreactors

Anca Roibu, Senne Fransen, M. Enis Leblebici, Glen Meir, Tom Van Gerven & Simon Kuhn*

KU Leuven, Department of Chemical Engineering, Celestijnenlaan 200F, 3001 Leuven, Belgium.
*Corresponding author: simon.kuhn@kuleuven.be

S1. Determination of the flow cell pathlength

According to the manufacturer specification the capillary in the cross-cell (Avantes) has an internal diameter of 0.075 cm. In order to determine the optical pathlength, we compared the absorbance of Rose Bengal (Sigma Aldrich) solutions in methanol (Fisher Scientific) of varying concentration acquired with a flow cell connected to a compact spectrometer (Ultra Low Straylight Fiber Optic UV/VIS/NIR spectrometer 200-1100 nm, Avantes) with the absorbance acquired with a spectrophotometer UV-Vis Lambda 365 (Perkin Elmer). We used Rose Bengal solutions due to their photostability during manipulation under environmental light in the laboratory. The absorbance measurements using the spectrophotometer UV-Vis Lambda 365 were realized with a resolution of 1 nm, scanning speed of 480 nm/scan and using a cuvette with an optical pathlength of 1 cm. The absorbance measurements using the UV-Vis flow cell connected to the compact spectrometer were realized at 1.3 ms integration time and 500 scans/measurement. Because no reference cell is connected to the compact spectrometer, a reference absorbance measurement realized by passing the solvent through the flow cell was performed after maximum two consecutive measurements. The concentration of the analyzed solutions ranged between 6 and $20\cdot10^{-6}$ M. The measurements were realized in 5 replicates. Supplementary Figure S1 illustrates the absorbance variation in function of concentration measured with the spectrophotometer and the flow cell respectively. In the calibration curve illustrated in Supplementary Fig. S1a the slope is equal to the molar absorption coefficient of Rose Bengal, ε_{RB} = 104252 $M^{-1}\,cm^{-1}$ (the optical pathlength is 1 cm). The optical pathlength of the flow cell can then be determined by dividing the slope observed in Supplementary Fig. S1b with ε_{RB}. We averaged the values found from five determinations and obtained an average optical pathlength of the flow cell equal to 0.0730 cm.

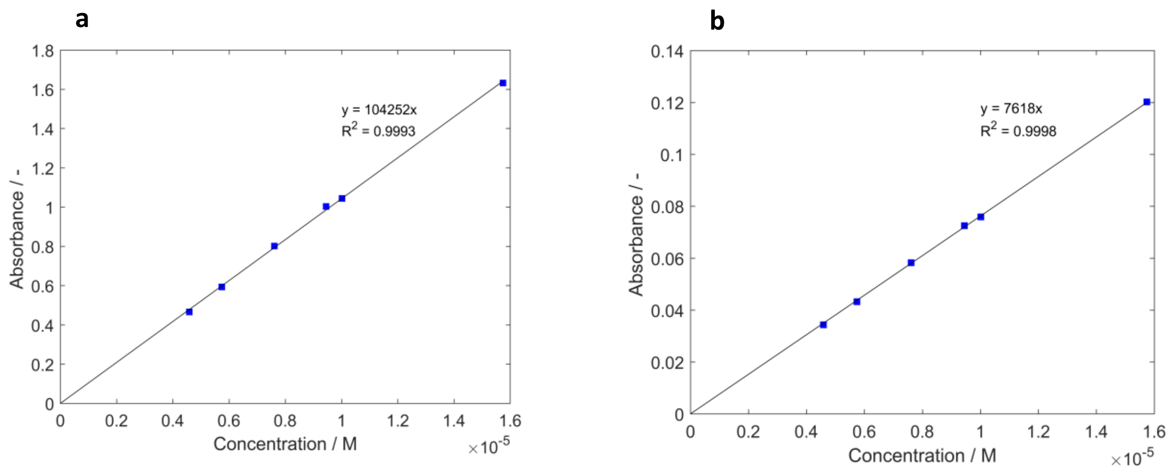

Supplementary Figure S1. (a) Absorbance at 557 nm in function of Rose Bengal concentration measured with UV-Vis Lambda 365 (Perkin Elmer), optical pathlength = 1 cm. Solvent: methanol. (b) Absorbance at 557-558 nm in function of Rose Bengal concentration measured with flow cell. Solvent: methanol.

S2. Calibration curve for DAE OF

Considering concentrations up to $0.5 \cdot 10^{-3}$ M, we determined a molar absorption coefficient of DAE OF (TCI Chemicals) equal to 28381 $M^{-1}cm^{-1}$ ± 248 $M^{-1}cm^{-1}$ from three measurements. Supplementary Fig. S2 illustrates an example of calibration curve used to determine the molar absorption coefficient of DAE OF.

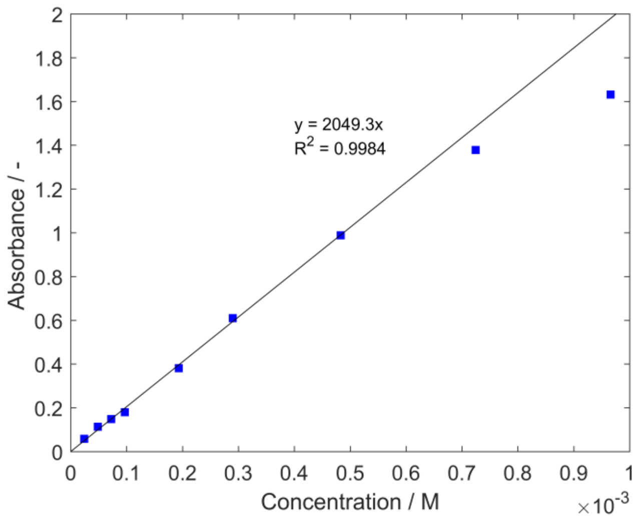

Supplementary Figure S2. Absorbance at 268-269 nm in function of DAE OF concentration measured with UV-Vis flow cell (optical pathlength = 0.073 cm). Solvent: hexane.

S3. Verification of DAE CF molar absorption coefficient

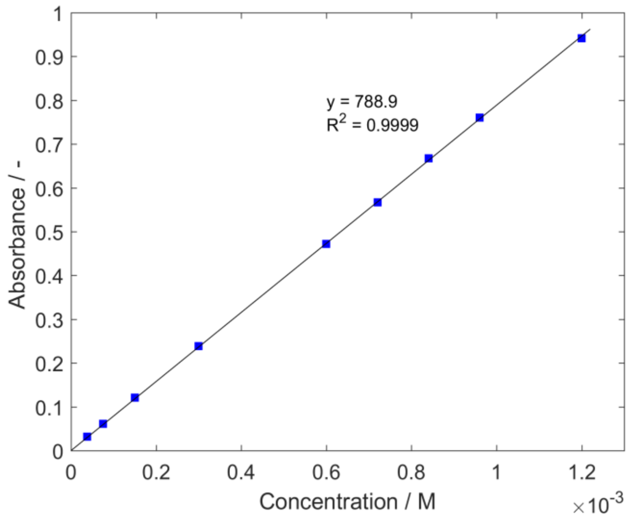

Supplementary Figure S3. Absorbance at 565.2-566.2 nm in function of DAE CF concentration measured using flow cell (optical pathlength = 0.073 cm). Solvent: hexane.

S4. Experimental set-up for generation of DAE CF

DAE CF was obtained by irradiation in continuous flow of DAE OF solution using the experimental set-up illustrated in Supplementary Fig.S4. The UV reactor consists of a capillary tube (PFA, internal diameter of 0.1 cm, length of around 100 cm) located on the top of an axial fan. The UV lamp is placed on the top of an enclosed box. The capillary tube and the fan are placed inside the box below the UV lamp. The DAE OF solution is fed through the reactor using a syringe pump and collected in a brown bottle. The fan is turned on in the case the period of continuous irradiation exceeds 1h.

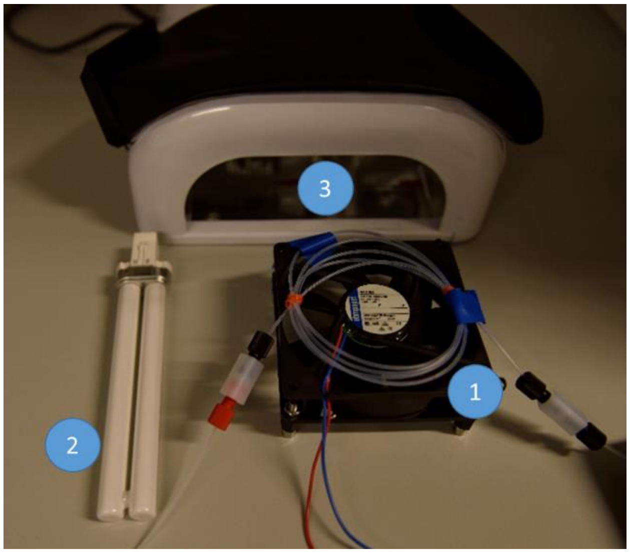

Supplementary Figure S4. Experimental set-up for production of DAE CF.
① Capillary tube placed on an axial fan
② UV lamp
③ Reactor box

S5. Experimental set-up for actinometric measurements

The experimental set-up pictured in Supplementary Fig. S5 is placed in a fume hood. The fume hood is covered with black fabric to avoid the exposure of the actinometer solutions to the exterior light. The syringe and the tubes are covered with black material during actinometric measurements, only the glass reactor being exposed to the radiation of the green LEDs. The syringe pump, the driver of the LEDs, the digital multimeter and the spectrometer are connected and controlled from a computer outside the ‘dark’ area.

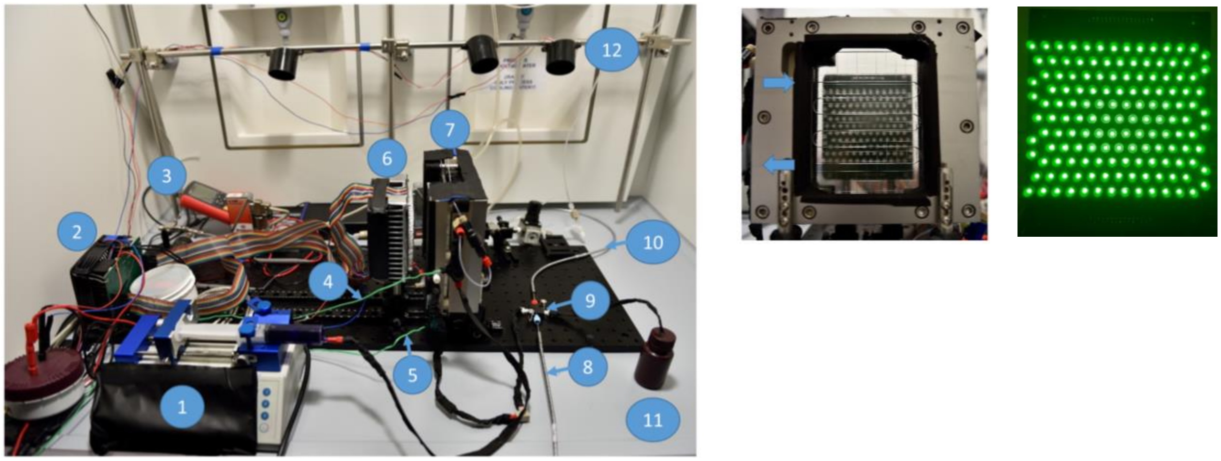

Supplementary Figure S5. Experimental set-up for actinometric measurements. The insets on the right side show the glass microreactor with its holder and the LED board.

① syringe pump
② driver for the LED board
③ digital multimeter
④ thermocouple (inlet)
⑤ thermocouple (outlet)
⑥ LED board
⑦ reactor
⑧ optical fiber, connects the lamp to the flow cell
⑨ flow cell
⑩ optical fiber, connects the flow cell to the spectrometer
⑪ collection bottle
⑫ red LEDs

S6. Spectral irradiance of the light sources

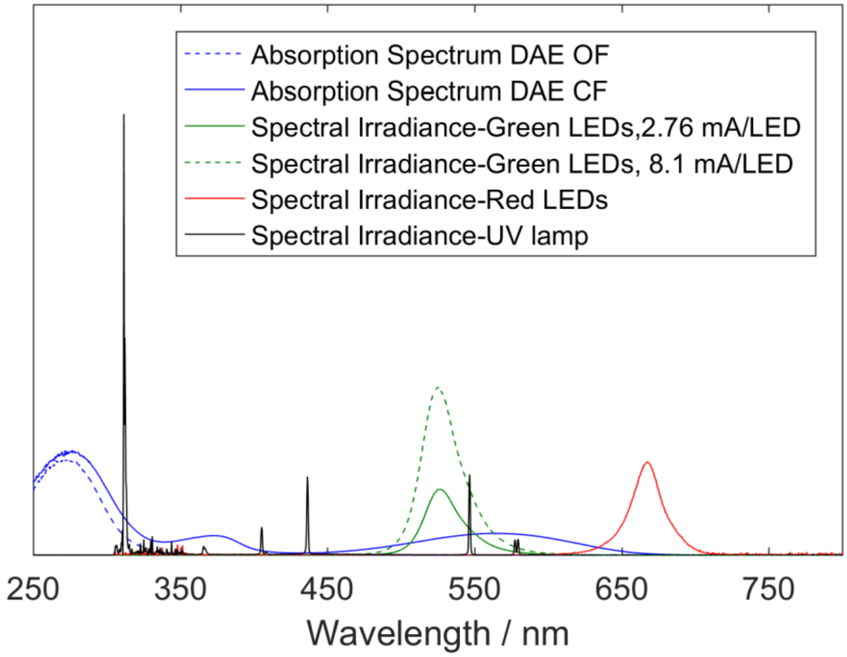

Supplementary Figure S6. Comparison between the absorption spectrum of DAE OF and DAE CF with the spectral irradiance of the light sources used in this work. The spectra are rescaled to allow a comparative analysis.

S7. Numerical integration procedure for polychromatic light

Upon irradiation with polychromatic light, the concentration of the DAE OF varies with the residence time in the photoreactor according to:

$$\frac{\mathrm{dc}}{\mathrm{dt}} = -\frac{I_0}{V}\sum_{\lambda=480}^{620}\phi_\lambda g_\lambda\left(1-10^{-\varepsilon_\lambda c(t) l_{\text{reactor}}}\right) \tag{S 1}$$

where c is the concentration of DAE CF [M], t [s] is residence time, I_0 [Einstein s^{-1}] is the photon flux received in the reactor, V [L] is the irradiated volume (reactor volume), ϕ_λ [mol $\mathrm{Einstein}^{-1}$] is the quantum yield of cycloreversion reaction, g_λ [-] is the density function of the light source, ε_λ [$M^{-1}cm^{-1}$] is the molar absorption coefficient and l_{reactor} [cm] is the light pathlength in the reactor.

In order to calculate the photon flux, we will first solve this equation numerically. As I_0 and V are constant in time, we define a new variable, τ, which is equal to:

$$\tau = t\frac{I_0}{V} \tag{S 2}$$

Hence, Supplementary equation (S 1) becomes

$$\frac{\mathrm{d}c}{\mathrm{d}\tau} = -\sum_{\lambda=480}^{620} \phi_\lambda g_\lambda \left(1 - 10^{-\varepsilon_\lambda c(\tau) l}\right) \quad \text{(S 3)}$$

The solver *ode45* provided by Matlab is used to integrate over a prescribed range of τ values, to determine the corresponding values of concentrations (c_{sim}). Next, the concentrations of DAE CF determined experimentally (c_{exp}) are interpolated with the curve of simulated concentrations (c_{sim}) in function of simulated τ values (τ_{sim}) to determine τ. From the experimental τ values, there are two approaches which can be followed. The first approach consists of calculating the photon flux from the slope ($\frac{I_0}{V}$) of the linear regression of τ in function of t. Next, I_0 is reintroduced in Supplementary equation (S1) which is then integrated numerically in order to obtain the concentration values at different residence times. As it can be observed in Supplementary Fig. S7d, the variation of concentration obtained using I_0 from the slope predicts the experimental data acquired at all residence times well. The second approach involves calculating the photon flux for each t. The photon flux determined at different residence times should be similar, if the output of the light source is stable in time. However, as it can be observed in Supplementary Fig. S7c, the photon flux increased by 5.4% from 1 to 10 ml min^{-1}. This could be due to the limited detection sensitivity or concentration and velocity gradients. At high flow rates the achieved conversions are small (below 4%), therefore the variation in absorbance which has to be registered is influenced by detection noise. For example, in the experiment using $3\cdot10^{-4}$ M concentration, the standard deviation of the detected absorbance represented 8% of the change in absorbance after 3 s ($\Delta A_{565\mathrm{nm}}$). In the case of residence time of 33 s the standard deviation was below 1% of $\Delta A_{565\mathrm{nm}}$. We calculated the variation of concentration of DAE CF using the photon flux determined from 3 s and 33 s residence time and compared with the value obtained from the slope. As it can be observed in Supplementary Fig. 7d, when using the photon flux determined at 33 s, the predicted concentrations are closed to the experimental data, in contrast with the predictions using photon flux from 3 s residence time which deviates from the experimental data at higher residence times.

Moreover, we observed that this increasing trend of photon flux with decreasing residence time can be sometimes reversed. An example for this case is illustrated by Supplementary Fig. S8. This observation shows that the variation of the photon flux with the residence time is mainly related to the detection.

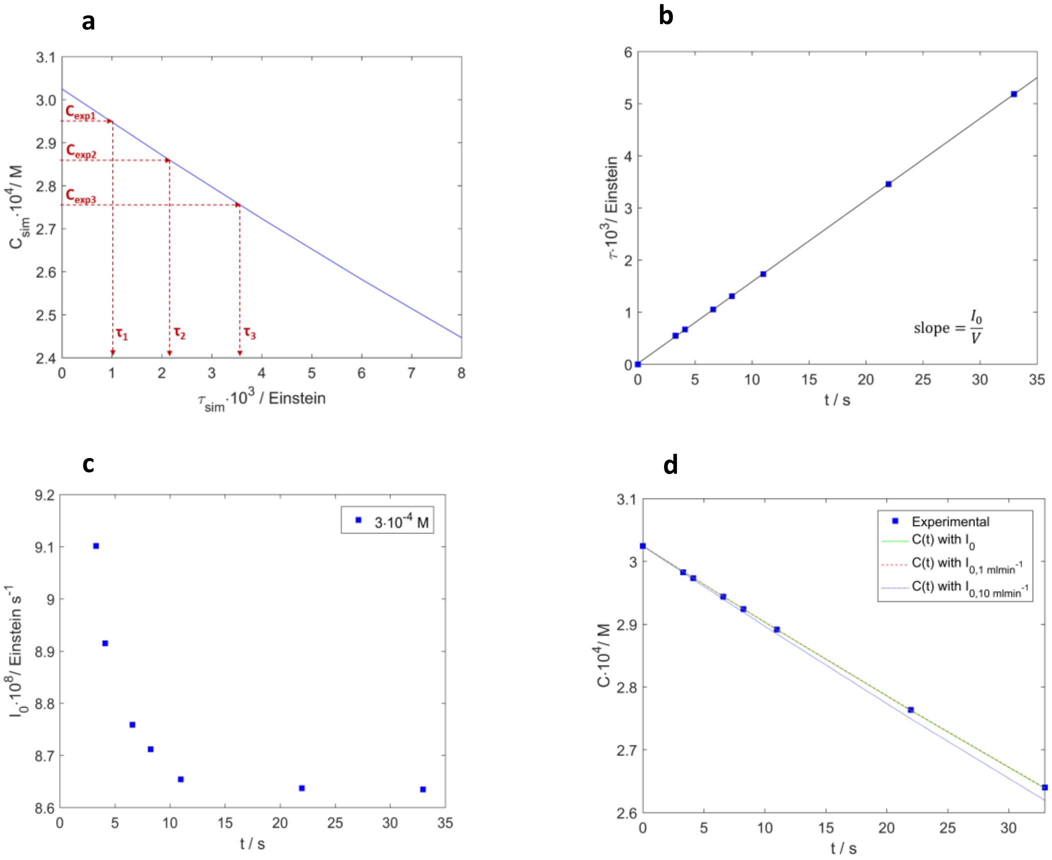

Supplementary Figure S7. Calculation procedures based on numerical integration for determining the photon flux. (a) Interpolation of DAE CF concentration experimentally determined (c_{exp}) for determining τ values. (b) Determination of photon flux (I_0) from the slope of τ vs. residence time (t). (c) Photon flux (I_0) calculated for each residence time, t. (d) Comparison between the observed variation of concentration (C) with residence time (t) and the predictions realized using photon flux determined from the slope, at 1 ml min^{-1} and 10 ml min^{-1} respectively. ($8.3 \cdot 10^{-4}$ M, 2 cm, $l_{reactor}$ = 0.0785 cm, I_F = 2.76 mA/LED).

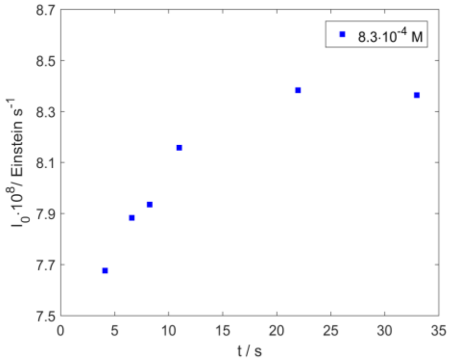

Supplementary Figure S8. Variation of the photon flux calculated using numerical integration with the residence time ($8.3 \cdot 10^{-4}$ M, 2 cm, $l_{reactor}$ = 0.0785 cm, I_F = 2.76 mA/LED).

S8. Mathematical derivation of the analytical solution

Upon irradiation with polychromatic light, the concentration of the DAE OF varies with the residence time in the photoreactor according to:

$$\frac{\mathrm{d}c}{\mathrm{d}t} = -\frac{I_0}{V}\sum_{\lambda=480}^{620}\phi_\lambda g_\lambda (1-10^{-\varepsilon_\lambda c(t) l_{\text{reactor}}}) \quad \text{(S 4)}$$

where c is the concentration of DAE CF [M], t [s] is residence time, I_0 [Einstein s^{-1}] is the photon flux received in the reactor, V [L] is the irradiated volume (reactor volume), ϕ_λ [mol Einstein^{-1}] is the quantum yield of cycloreversion reaction, g_λ [-] is the density function of the light source, ε_λ [$M^{-1}cm^{-1}$] is the molar absorption coefficient and l_{reactor} [cm] is the light pathlength in the reactor.

Using e as the base of the exponentiation:

$$\frac{\mathrm{d}c}{\mathrm{d}t} = -\frac{I_0}{V}\sum_{\lambda=480}^{620}\phi_\lambda g_\lambda \left[1-\exp\left(-\varepsilon'_\lambda c(t) l_{\text{reactor}}\right)\right] \quad \text{(S 5)}$$

with $\varepsilon'_\lambda = \varepsilon_\lambda \ln(10)$ (S 6)

$$\frac{\mathrm{d}c}{\mathrm{d}t} = -\frac{I_0}{V}\sum_{\lambda=480}^{620}\phi_\lambda g_\lambda + \frac{I_0}{V}\sum_{\lambda=480}^{620}\phi_\lambda g_\lambda \exp\left(-\varepsilon'_\lambda c(t) l_{\text{reactor}}\right) \quad \text{(S 7)}$$

In order to reduce the number of independent variables, consider the following substitutions:

$$y = \left(\sum_{\lambda=480}^{620}\varepsilon'_\lambda g_\lambda\right) c(t) l_{\text{reactor}} \quad \text{(S 8)}$$

$$x = \left(\sum_{\lambda=480}^{620}\phi_\lambda g_\lambda \varepsilon'_\lambda\right) l_{\text{reactor}} \frac{I_0}{V} t \quad \text{(S 9)}$$

$$\frac{\mathrm{d}y}{\mathrm{d}x} = \frac{\left(\sum_{\lambda=480}^{620}\varepsilon'_\lambda g_\lambda\right) l_{\text{reactor}} V}{\left(\sum_{\lambda=480}^{620}\phi_\lambda g_\lambda \varepsilon'_\lambda\right) l_{\text{reactor}} I_0} \frac{\mathrm{d}c}{\mathrm{d}t} \quad \text{(S 10)}$$

Therefore, the obtained Supplementary equations (S8) and (S10) are used to rewrite the Supplementary equation (S7) as:

$$\frac{\mathrm{d}y}{\mathrm{d}x} = \frac{\left(\sum_{\lambda=480}^{620}\varepsilon'_\lambda g_\lambda\right) V}{\left(\sum_{\lambda=480}^{620}\phi_\lambda g_\lambda \varepsilon'_\lambda\right) I_0}\left[-\frac{I_0}{V}\sum_{\lambda=480}^{620}\phi_\lambda g_\lambda + \frac{I_0}{V}\sum_{\lambda=480}^{620}\phi_\lambda g_\lambda \exp\left(-\frac{\varepsilon'_\lambda y}{\sum_{\lambda=480}^{620}\varepsilon'_\lambda g_\lambda}\right)\right] \quad \text{(S 11)}$$

Define the parameter δ_λ as:

$$\delta_\lambda = \frac{\varepsilon_\lambda^{'}}{\sum_{\lambda=480}^{620} \varepsilon_\lambda^{'} g_\lambda} - 1 \tag{S 12}$$

Hence, the Supplementary equation (S11) becomes

$$\frac{\mathrm{d}y}{\mathrm{d}x} = \frac{\left(\sum_{\lambda=480}^{620} \varepsilon_\lambda^{'} g_\lambda\right)}{\left(\sum_{\lambda=480}^{620} \phi_\lambda g_\lambda \varepsilon_\lambda^{'}\right)} \left[-\sum_{\lambda=480}^{620} \phi_\lambda g_\lambda + \sum_{\lambda=480}^{620} \phi_\lambda g_\lambda \exp(-y(\delta_\lambda + 1)) \right] \tag{S 13}$$

Note that $a^{(x+1)}$ can be written as a Maclaurin series:

$$a^{x+1} = a + ax\ln(a) + \frac{ax^2}{2}\ln^2(a) + \ldots \tag{S 14}$$

Hence for $\delta \to 0$, $\exp[-y(1+\delta_\lambda)]$ can be approximated by:

$$[\exp(-y)]^{1+\delta_\lambda} \approx \exp(-y) - \exp(-y) y \delta_\lambda \tag{S 15}$$

Substituting this approximation into Supplementary equation (S13) gives

$$\frac{dy}{dx} = \frac{\left(\sum_{\lambda=480}^{620} \varepsilon_\lambda^{'} g_\lambda\right)}{\left(\sum_{\lambda=480}^{620} \phi_\lambda g_\lambda \varepsilon_\lambda^{'}\right)} \left[-\sum_{\lambda=480}^{620} \phi_\lambda g_\lambda + \sum_{\lambda=480}^{620} \phi_\lambda g_\lambda \left(\exp(-y) - \exp(-y) y \delta_\lambda\right) \right] \tag{S 16}$$

or equivalently

$$\frac{\mathrm{d}y}{\mathrm{d}x} = \frac{\left(\sum_{\lambda=480}^{620} \varepsilon_\lambda^{'} g_\lambda\right)\left(\sum_{\lambda=480}^{620} \phi_\lambda g_\lambda\right)}{\left(\sum_{\lambda=480}^{620} \phi_\lambda g_\lambda \varepsilon_\lambda^{'}\right)} \left[-1 + \exp(-y) - \frac{\sum_{\lambda=480}^{620} \phi_\lambda g_\lambda \delta_\lambda \left(\exp(-y) y\right)}{\left(\sum_{\lambda=480}^{620} \phi_\lambda g_\lambda\right)} \right] \tag{S 17}$$

Define the factors *f* and α, so that the Supplementary equation (S17) can be written more concisely.

$$f = \frac{\left(\sum_{\lambda=480}^{620} \varepsilon'_\lambda g_\lambda\right)\left(\sum_{\lambda=480}^{620} \phi_\lambda g_\lambda\right)}{\left(\sum_{\lambda=480}^{620} \phi_\lambda g_\lambda \varepsilon'_\lambda\right)} \tag{S 18}$$

$$\alpha = \frac{\left(\sum_{\lambda=480}^{620} \phi_\lambda g_\lambda \delta_\lambda\right)}{\left(\sum_{\lambda=480}^{620} \phi_\lambda g_\lambda\right)} \tag{S 19}$$

$$\frac{\mathrm{d}y}{\mathrm{d}x} = f\left[-1 + \exp(-y) - \alpha \exp(-y) y\right] \tag{S 20}$$

As δ_λ approaches 0, α will approach 0 as well. For our case, $|\alpha| = 0.01$. Hence, as a first approximation, we set $\alpha = 0$ in Supplementary equation (S20).

$$\frac{\mathrm{d}y}{\mathrm{d}x} \approx f\left[-1 + \exp(-y)\right] \tag{S 21}$$

The solution of Supplementary equation (S21) is completely analogous to the solution of Supplementary equation (S4) for monochromatic light. Hence

$$y \approx \ln\left\{1 + \left[\exp(y(0)) - 1\right]\exp(-fx)\right\} \tag{S 22}$$

with $y(0)$ equal to equation (S8), evaluated for the initial concentration $c(0)$.

Formally, this is the 0th order term in an asymptotic series

$$y = y_0 + \alpha y_1 + \alpha^2 y_2 + \ldots \tag{S 23}$$

However, as Supplementary equation (S22) agrees very well with the numerical solution, we will not develop this further.

By returning to the initial variables, Supplementary equation (S22) becomes:

$$\left(\sum_{\lambda=480}^{620} \varepsilon'_\lambda g_\lambda\right) c(t) l = \ln\left\{1 + \left[\exp\left(\left(\sum_{\lambda=480}^{620} \varepsilon'_\lambda g_\lambda\right) c(0) l_{\text{reactor}}\right) - 1\right] \exp\left[-\left(\sum_{\lambda=480}^{620} \varepsilon'_\lambda g_\lambda\right)\left(\sum_{\lambda=480}^{620} \phi_\lambda g_\lambda\right) l_{\text{reactor}} \frac{I_0}{V} t\right]\right\} \tag{S 24}$$

Define $\varepsilon'_{\text{avg}}$ and ϕ_{avg} as:

$$\varepsilon'_{\text{avg}} = \sum_{\lambda=480}^{620} \varepsilon'_\lambda g_\lambda \tag{S 25}$$

$$\phi_{avg} = \sum_{\lambda=480}^{620} \phi_\lambda g_\lambda \tag{S 26}$$

The variation of concentration with residence time $c(t)$ can be computed as:

$$c(t)=\frac{1}{\varepsilon'_{\text{avg}} l_{\text{reactor}}}\ln\left\{1+\left[\exp\left(\varepsilon'_{\text{avg}} c(0) l_{\text{reactor}}\right)-1\right]\exp\left(-\varepsilon'_{\text{avg}}\phi_{\text{avg}} l_{\text{reactor}}\frac{I_0}{V}t\right)\right\} \quad \text{(S 27)}$$

The photon flux can be determined using

$$\ln\left[\exp\left(\varepsilon'_{\text{avg}} c(0) l_{\text{reactor}}\right)-1\right]-\ln\left[\exp\left(\varepsilon'_{\text{avg}} c(t) l_{\text{reactor}}\right)-1\right]=\varepsilon'_{\text{avg}}\phi_{\text{avg}} l_{\text{reactor}}\frac{I_0}{V}t \quad \text{(S 28)}$$

Alternatively, when the variable ε'_{λ} defined in Supplementary equation (S6) is replaced with the molar absorption coefficient, ε_{λ}, and 10 is the base of exponentiation, Supplementary equations (S27) and (S28) become:

$$\log\left(10^{\varepsilon_{\text{avg}} c(t) l_{\text{reactor}}}-1\right)-\log\left(10^{\varepsilon_{\text{avg}} c(0) l_{\text{reactor}}}-1\right)=-\varepsilon_{\text{avg}}\phi_{\text{avg}} l_{\text{reactor}}\frac{I_0}{V}t \quad \text{(S 29)}$$

$$c(t)=\frac{1}{\varepsilon_{\text{avg}} l_{\text{reactor}}}\log\left[1+\left(10^{\varepsilon_{\text{avg}} c(0) l_{\text{reactor}}}-1\right)10^{-\varepsilon_{\text{avg}}\phi_{\text{avg}} l_{\text{reactor}}\frac{I_0}{V}t}\right] \quad \text{(S 30)}$$

S9. Perfect mixing vs. influence of diffusion limitations

Performing actinometric measurements requires perfect mixing. This condition is easily achieved in stirred batch systems. In microreactors, mixing is not achieved by active means and is therefore diffusion limited. To check the presence of concentration gradients at the outlet of the microreactor, Aillet et al.[1] proposed the calculation of Fourier number, Fo, and the Damköhler II number, Da_{II}. Fo is defined as the ratio between the residence time, *t*, and the radial diffusion time, t_d [s]:

$$Fo=\frac{t}{t_d} \quad \text{(S 31)}$$

with

$$t=\frac{V}{Q} \quad \text{(S 32)}$$

$$t_d=\frac{d^2}{D_m} \quad \text{(S 33)}$$

Where *V* [mL] is the irradiated volume (0.5494 mL), *Q* is the volumetric flow rate [ml min^{-1}], *d* [cm] is the diameter of the microreactor channel and D_m is the molecular diffusion coefficient (assumed as 10^{-5} cm^2 s^{-1})[1]. Due to the parabolic velocity profile in laminar flow, the molecules which are closer to the microreactor wall are irradiated for a longer time than the molecules in the center. Therefore, the molecules will be homogeneously distributed at the outlet of the microreactor, if the diffusion time in radial direction is smaller than the residence time (Fo ≥ 1).

Da_{II} is defined as the ratio between the radial diffusion time and the characteristic reaction time[1]:

$$Da_{II} = \frac{t_d}{t_r} \quad \text{(S 34)}$$

with $$t_r = \frac{c(0)}{r} = \frac{c(0)}{\frac{I_{0,\lambda}}{V}\sum_{\lambda=480}^{620} \phi_\lambda g_\lambda \left(1-10^{-\frac{\varepsilon_\lambda c(0) l_{reactor}}{2}}\right)} \quad \text{(S 35)}$$

where t_r [s] is the characteristic time of the reaction as reported by Aillet et al.[1] When $Da_{II} < 1$, the time necessary for the molecules to diffuse across the reactor channel is shorter than the characteristic time of the reaction, therefore the concentration will be homogeneous in radial direction at the outlet of the microreactor[1].

Fo and Da_{II} were calculated for our system considering a photon flux of $7.9\cdot10^{-8}$ Einstein s^{-1} and a pathlength of 0.0889 cm. Fo was found to range from $3.3\cdot10^{-3}$ at 3 s residence time to $3.3\cdot10^{-1}$ at 330 s. Da_{II} increased from 1.6 at $11.7\cdot10^{-4}$ M to 2.3 at $3\cdot10^{-4}$ M. In order to achieve Fo ≥ 1, we should perform the measurements at flow rates smaller than 0.03 $mlmin^{-1}$ and to achieve $Da_{II} < 1$ solutions of $25\cdot10^{-4}$ M should be used. However, these conditions are difficult to achieve due to decreased accuracy of the syringe pumps at low flow rates and on-line detection limitations. However, as depicted in Fig. 4b-c we have not observed the effects of the concentration gradients in our operation conditions. In order to verify if the diffusion limitations will affect the output at an increased value of Da_{II} = 6.8 we have performed actinometric measurements at $3\cdot10^{-4}$ M and at forward current, I_F, of 8.1 mA/LED. The good prediction of the concentration variation is in accordance with the findings of Aillet al al.[1], who reported that in the case of a single absorbing species the diffusion limitations ($Da_{II} > 1$) are not affecting the conversion measured at the microreactor outlet.

References

1 Aillet, T., Loubière, K., Prat, L. & Dechy-Cabaret, O. Impact of the diffusion limitation in microphotoreactors. *AIChE J.* **61**, 1284-1299 (2015).
